# Epidemic risk from friendship network data: an equivalence with a non-uniform sampling of contact networks

**DOI:** 10.1038/srep24593

**Published:** 2016-04-15

**Authors:** Julie Fournet, Alain Barrat

**Affiliations:** 1 Aix Marseille Université, Université de Toulon, CNRS, CPT, UMR 7332, 13288, Marseille, France; 2 Data Science Laboratory, ISI Foundation, Torino, Italy

## Abstract

Contacts between individuals play an important role in determining how infectious diseases spread. Various methods to gather data on such contacts co-exist, from surveys to wearable sensors. Comparisons of data obtained by different methods in the same context are however scarce, in particular with respect to their use in data-driven models of spreading processes. Here, we use a combined data set describing contacts registered by sensors and friendship relations in the same population to address this issue in a case study. We investigate if the use of the friendship network is equivalent to a sampling procedure performed on the sensor contact network with respect to the outcome of simulations of spreading processes: such an equivalence might indeed give hints on ways to compensate for the incompleteness of contact data deduced from surveys. We show that this is indeed the case for these data, for a specifically designed sampling procedure, in which respondents report their neighbors with a probability depending on their contact time. We study the impact of this specific sampling procedure on several data sets, discuss limitations of our approach and its possible applications in the use of data sets of various origins in data-driven simulations of epidemic processes.

Contact patterns between individuals, and in particular face-to-face interactions, play an essential role in determining how infectious diseases spread within a population. Information about these patterns is therefore crucial to inform epidemic models and ensure relevant predictions. Many efforts have been made to collect data about contacts between individuals using different techniques^1^,^2^,^3^. In particular, technological advances make it now possible to collect accurate data on face-to-face contacts with a high spatial and temporal resolution in various contexts, by using infrastructures based on wearable sensors^3^,^4^,^5^,^6^,^7^,^8^,^9^,^10^.

Such infrastructures are typically deployed in specific environments (schools, hospitals, etc.) and for limited time windows (typically few days or weeks). Moreover, even if their use has recently become more widespread, as costs have decreased and different groups have developed similar tools, they cannot always nor systematically be used in any context. Other types of data, such as surveys, diaries or online interactions, which have long been used in epidemiological contexts^1^,^2^, remain thus important sources that feed models of epidemic spread. Data on contacts between individuals coming from different types of sources are however not equivalent, as explored quantitatively in recent studies^11^,^12^. For instance, contact diaries lead to an underestimation of the number of actual contacts, as short contacts are not well reported, together with an overestimation of the duration of contacts^11^,^12^. In this respect, friendship surveys, in which participants are asked to name their friends, might be less affected by memory biases; however, the precise relation between the network of friendships and the network of contacts is not straightforward, even in a given context, as friends might meet relatively rarely while many daily encounters occur between non-friends. For instance, ref. 11 reports on a high school context, where data was collected both on actual contacts between students of 9 classes using wearable sensors, and on their friendship relations through a survey. It was found that (i) the friendship survey suffered from low participation rate, (ii) the longest contacts corresponded to reported friendships and most friendship relations lead to actual face-to-face encounters, but (iii) many short contacts did not correspond to reported friendships, resulting in a friendship network with much lower density than the contact network.

Here, we investigate further questions arising from this comparison. First, we assess how these differences between the two networks of interactions (reported friendships vs. measured contacts) lead to different outcomes if they are used as substrate for possible propagation events in numerical simulations of spreading processes. Indeed, although the longest contacts are reported in the friendship survey, the non-reported many short contacts can have an important role in propagation processes. Second, we ask if using the reported friendship network is equivalent, with respect to the outcome of such simulations, to a specific sampling of the contact network. Various sampling procedures are known to affect networks’ measured properties in different ways, and many works have studied for instance how average degree, degree distribution, clustering or assortativity properties depend on the procedure and on the sample size^13^,^14^,^15^,^16^,^17^,^18^,^19^. Fewer studies have investigated how the outcome of simulations of dynamical processes in data-driven models is affected if incomplete data are used^20^,^21^,^22^,^23^. As incomplete network data are in fact quite common, researchers have moreover tackled the issue of inferring network statistics^24^,^25^,^26^,^27^ from incomplete data. Since many networks are the support of dynamical processes, it is also crucial to develop methods to obtain estimates of the outcome of such processes in the case of sampled data^21^,^23^. In this perspective, understanding if the differences in outcomes between contact and friendship data may be seen as biases due to a sampling process might then give hints on how to compensate for such biases and how to use the information contained in the friendship network to obtain accurate prediction on the epidemic risk, even in the absence of data on the actual contact network, in the spirit of ref. 23.

To make progresses in these directions, we rely here on the case study of a publicly available data set containing information on contacts measured by wearable sensors as well as friendship relations in the same population, namely high school students^11^. This is indeed to the best of our knowledge the only available data set combining contacts and friendship data in a given population. We first show how simulations of the spread of infectious diseases using friendship data lead to a strong underestimation of the epidemic risk with respect to using the contact network measured using wearable sensors, as can be expected due to the lower participation rate and the absence of many weak links in the friendship surveys. We then consider several sampling procedures on the contact network and compare the outcome of simulations on the sampled networks and on the friendship network, for different values of the spreading parameters. Simple sampling methods such as uniform random sampling of nodes or edges do not reproduce the outcome of simulations using the friendship survey. We then put forward a non-uniform sampling method that favors sampling of the most important contacts of each sampled individual, and show that the outcome of spreading processes on the resulting sampled networks is equivalent to the one obtained when the friendship network is considered. Note that at this stage we do not use the friendship network to provide an estimate of the real epidemic risk in the contact network, but rather assess how using friendship data would be similar to a bias due to a specific sampling method on the real contact network. We finally apply the sampling method to several data sets and study how changing its parameters (number of nodes sampled, density of sampled network) impacts the outcome of spreading simulations.

## Results

### Data and methodology

We consider data collected and made publicly available by the SocioPatterns collaboration, which describe two types of social interactions, namely face-to-face contacts and friendship relations, between the same individuals, namely students in a high school (see the SocioPatterns website http://www.sociopatterns.org/). The contact network, measured using wearable sensors, has 327 nodes and 5818 weighted edges. Each edge (*i*¸*j*) with weight *W*_*ij*_ corresponds to the fact that individuals *i* and *j* have been in contact for a total time *W*_*ij*_ during the deployment, which lasted one week. The friendship network, obtained through a survey, has *N*_*F*_ = 135 nodes and *E*_*F*_ = 413 unweighted edges. All the nodes of the friendship network (but one) belong to the contact network. We also note that the population under study was structured in 9 classes (see Methods).

While Mastrandrea *et al.*^11^ performed a quantitative comparison of the contact and friendship networks, we are here interested in comparing the use of these networks within the context of models of propagation phenomena. To this aim, we consider the paradigmatic Susceptible-Infectious-Recovered (SIR) model: in this model, a Susceptible (S) node *i* in contact with an Infectious one *j* can become Infectious (I). If the edge between *i* and *j* has weight *W*_*ij*_, the rate at which this event occurs is given by *βW*_*ij*_/*T* where *T* is the total measurement time (see Methods and Stehlé *et al.*^28^ for details). Each Infectious node becomes Recovered (R) at rate *μ* and cannot be infected anymore. The process ends when there are no Infectious nodes any more. We perform numerical simulations of this model on the contact network, on the friendship network and on networks obtained by sampling the contact network using several sampling methods (described in the next paragraph). To quantify the epidemic risk and compare outcomes of these simulations, we measure the distributions of epidemic sizes (i.e., the final fraction of recovered nodes), the fraction of epidemics with size larger than 20% and the average size of these epidemics (the cut-off of 20% is chosen arbitrarily to distinguish between small and large epidemics; changing the value of this threshold does not alter our results).

It is important to note that we consider here a static version of the contact network, while the data at hand provides temporally resolved contacts. The rationale behind this choice is twofold. First, the friendship survey data does not contain temporal information. If using friendship network can be seen as a sampling of contact networks, it is thus necessarily a static sample. Second, when modeling the propagation of infectious diseases with realistic timescales of several days, it has been shown in^28^ that a static weighted contact network contains enough information to obtain a good estimate of the process outcome. Clearly, when dealing with faster processes, the temporal evolution of the network becomes relevant; in that case, studies such as^23^ have shown how to build realistic surrogate timelines of contacts on weighted networks, using the robustness of the distributions of the durations of single contact events and of the intervals between successive contacts measured in different contexts.

### Sampling methods

**Figure 1:**
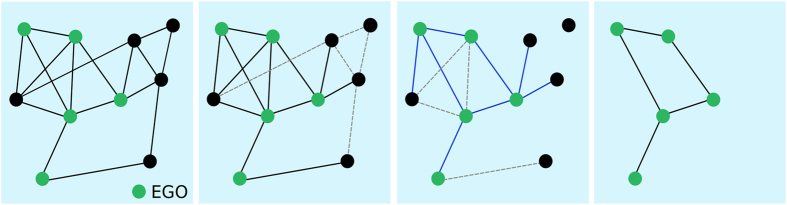
Sketch of the EGOref sampling process. We first select a certain number of nodes as egos (in green), who represent the respondents to the surveys. Links between non-respondents cannot be observed (dashed grey links in the second panel). Each ego then “chooses” to report some of its links, with probability depending on their weights (links shown in blue in the third panel, while the non-reported links are shown in dashed grey lines). We then finally keep only the egos and, among the chosen edges, only the ones joining egos (last panel).

Many different sampling procedures of network data have been considered in previous works, and their impact on the network’s statistical properties have been studied^13^,^14^,^15^,^16^,^17^,^18^,^19^. Sampling of the network used as substrate for transmission events is also known to affect the result of simulations of epidemic spread^20^,^21^,^23^. In particular, population sampling has a strong impact, even in the case of uniform sampling^23^. We therefore consider here the following sampling procedures on the contact network, tuned to obtain the same number of nodes as in the friendship network:

- We first consider as reference the Subgraph method (“SubFr”): we consider the 134 nodes of the Friendship network present in the contact network and take the subgraph induced by these nodes on the contact network. This would correspond to a population sampling of the contact network, with the sampled population corresponding to the respondents of the friendship survey.
- In the Random Node method (“RN”), we choose *N*_*F*_ = 135 nodes uniformly at random from the contact network and we take the subgraph induced by these nodes on the contact network. This corresponds to a population sampling with uniformly random choice of the sampled nodes.
- We also consider the Egocentric sampling method (“EGO”): we select a node at random and include this node and all its neighbors in the sample. We repeat this step until we reach the desired number of nodes, *N*_*F*_. If this number is exceeded by including the chosen node and its neighbors, we randomly choose a set of its neighbors so that the right number of nodes is exactly reached. Then, we take the subgraph induced by this sample of nodes on the original network.
- As these methods do not allow us to control the number of edges in the sampled network, we also consider several additional sampling methods, in which we can tune this number and set it equal to *E*_*_F_*_.
  - In the Random Edge method (“RE”), we first choose edges at random from the contact network until we reach the desired number of nodes *N*_*F*_; as the number of chosen edges is still lower than *E*_*F*_ we then choose edges at random from the contact network with the condition that both their extremities are in the set of nodes obtained in the first step, until the desired number of edges is reached.
  - In the Refined Random Node method (“RNref”), we add the following step to the RN method: after the subgraph is obtained, we remove edges at random to get the desired number of edges in the final sampled network.
  - We propose a new Refined Egocentric method (“EGOref”), inspired by the result of ref. 11 that the longest contacts corresponded to reported friendships, while many short contacts did not. Here, we select *N* nodes called *egos* at random from the contact network. For each *ego i* we select some of its edges as follows: each edge (i,j) is selected with a probability equal to 
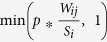
, with *W*_*ij*_ the weight of the edge between *i* and *j*, 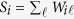 the strength of the *ego* node *i* and *p* is the parameter of the model. We then keep only the *egos* and the selected edges linking them and we remove the other edges (between egos and non-egos) and nodes (non-egos). With this method, we end up with the desired number of nodes by setting *N* = *N*_*F*_, and a number of edges that depends on the parameter *p*. Figure 1 summarizes this process.
  - While the *egos* are chosen uniformly at random among the nodes of the contact network in the EGOref method, we also consider a heterogenous EGOref method (“EGOref-het”), in which the distribution of *egos* in the various high school classes corresponds to the one of the friendship network (*egos* still being chosen at random within each class).

When using the sampled contact networks and the friendship network to simulate SIR processes, we moreover assign to each sampled edge a weight taken at random from the empirical distribution of weights of the contact network, which is known to be a robust feature of human contact patterns^3^,^29^ (See also the Supplementary Information for more details about the assignment of weights to links).

### Properties of sampled networks and outcomes of SIR simulations

Table 1 shows the characteristics of the empirical contact and friendship networks compared to the networks obtained by the simplest sampling techniques (SubFr, RN, EGO, RE). The contact network has 327 nodes, while the friendship and sampled networks have 135 nodes. The density is twice higher in the contact network than in the friendship network; however, the subgraph induced by the nodes of the friendship network (SubFr) has in fact a slightly higher density than the contact network. As the RN method samples uniformly the nodes of the contact network, the resulting density is on average equal to the density of the contact network. On the other hand, the density of the EGO sampled networks is even higher. Finally, RE sampling yields by construction the same density as the friendship network.

The clustering coefficient displays interesting features: despite a much lower density, the friendship network has a higher clustering coefficient than the contact network. Networks obtained through the SubFr, RN and EGO methods have as well rather large clustering coefficients, while the RE sampling yields much lower values.

**Figure 2:**
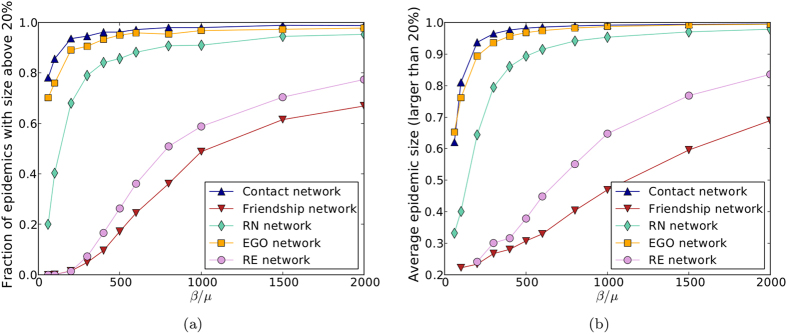
Outcome of SIR spreading simulations performed on empirical and sampled networks. (**a**) Fraction of epidemics with size above 20% (at least 20% of recovered individuals at the end of the SIR process) as a function of *β*/*μ*. (**b**) Average size of epidemic with size above 20% as a function of the parameter of spreading *β*/*μ*.

Figure 2 shows the outcome of the spreading simulations performed on the two empirical networks and on sampled networks obtained by the RN, RE and EGO sampling methods: it displays the fraction of epidemics with size above 20% and the average size of epidemics among the ones with size above 20%, as a function of the spreading parameter *β*/*μ*. As expected, simulations using the friendship network give a very strong underestimation of the epidemic risk with respect to the ones using the contact network. Moreover, simulations performed on the sampled networks RN and EGO yield a much larger estimation of the epidemic risk than when using the friendship network, and this estimation increases as the density of the network obtained by sampling increases (as is expected since the availability of transmission paths increases). Finally, simulations using the RE sampled networks also yield larger estimated epidemic sizes than when using the friendship network, although these networks have the same density. This is in agreement with the results of Smieszek *et al.*^30^ stating that high clustering values tend to hinder propagation processes, at fixed density: the clustering coefficient of the RE networks is indeed much smaller than the one of the friendship network.

We show in the Supplementary Information the results of simulations performed on the SubFr network as well as on a randomized version of the friendship network using the rewiring algorithm of Maslov *et al.*^31^ (MSZ). The SubFr network corresponds to a population sampling of the contact network, and leads to a limited underestimation of the epidemic risk with respect to the whole contact network. The MSZ network has the same number of nodes and edges than the frienship network, hence the same density, but a much smaller clustering, due to the randomization, and gives thus a higher epidemic risk.

**Figure 3:**
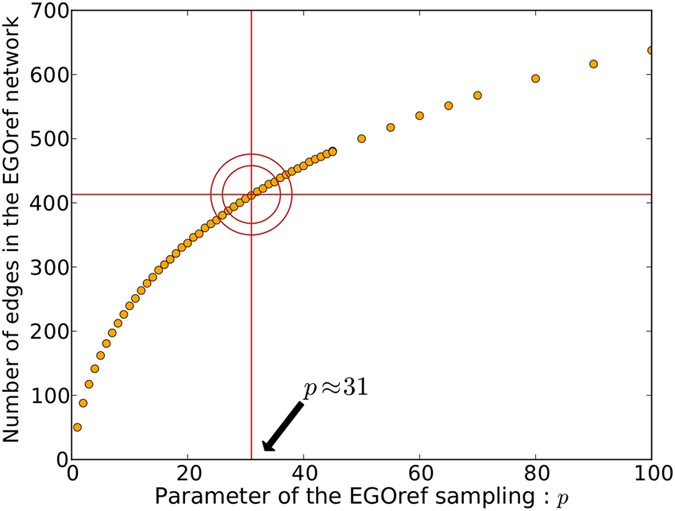
Average number of edges in the sampled network obtained by the EGOref method as a function of the parameter *p* with *N*_*F*_ = 135. The horizontal red line represents the number of edges *E*_*F*_ in the friendship network.

We now turn to the case of the EGOref sampling method. In the resulting sampled networks, the number of edges depends on the parameter *p*, at fixed number of sampled nodes *N*_*F*_. Figure 3 shows the average number of edges in the sampled network as a function of *p*. For *p* > max_*i*,*j*_(*s*_*i*_/*W*_*ij*_), this number reaches the average number of edges in the subgraph induced by *N*_*F*_ nodes chosen at random on the contact network, which is equal to the number of edges obtained through the RN sampling process. As our goal is to obtain a sampled subgraph of the contact network that is similar to the friendship network, we tune *p* in order to obtain sampled networks with an averaged number of edges close to *E*_*F*_ = 413, the number of edges in the friendship network. This value is obtained for *p* ≈ 31.3 (Fig. 3). At this value, the obtained network results always connected.

Table 2 compares the properties of the contact networks sampled by the RE, RNref and EGOref methods, in which we tune the number of edges, with the friendship network. Both average shortest path and clustering coefficient are closest to the friendship network case for the EGOref method, but the clustering remains in all cases smaller in the sampled networks than in the friendship network. Further refinements of the EGOref method might yield a clustering closer to the one of the friendship network, at the cost however of an increase in the method’s complexity and number of parameters.

**Figure 4:**
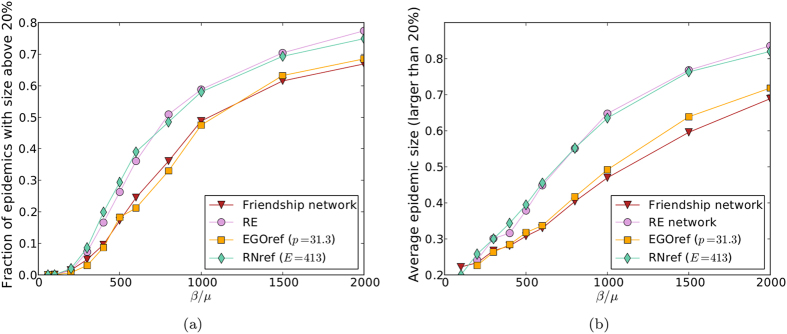
Outcome of SIR spreading simulations performed on friendship and sampled networks. (**a**) Fraction of epidemics with size above 20% (at least 20% of recovered individuals at the end of the SIR process) as a function of the parameter of spreading *β*/*μ*. (**b**) Average size of epidemic with size above 20% as a function of the spreading parameter *β*/*μ*.

Figure 4 shows the outcomes of epidemic spreading simulations performed on the friendship network and on the contact networks sampled using the EGOref, RNref and RE methods. A very good agreement with the epidemic risk estimated from the friendship network is obtained for the EGOref sampling, while the RE and RNref sampled networks yield large epidemic sizes with higher probability and larger average epidemic sizes, even if they have the same density. As mentioned above, this could be expected given their smaller clustering.

**Figure 5:**
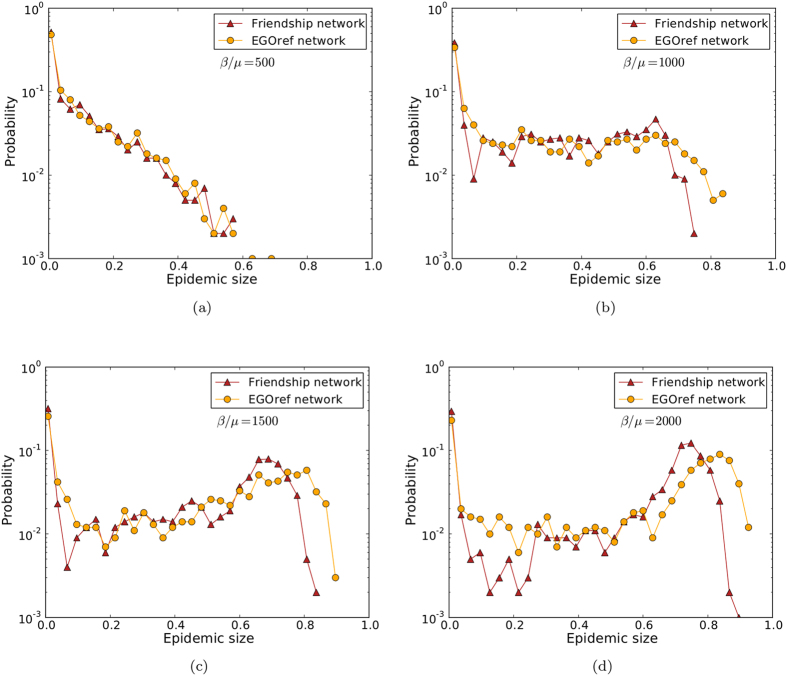
Distributions of epidemic sizes of SIR spreading simulations. We compare the outcomes of SIR spreading simulations performed on friendship and EGOref networks for different values of *β*/*μ*.

Figure 5 displays the whole distributions of epidemic sizes for simulations performed on the friendship and EGOref networks, for 4 values of the spreading parameter *β*/*μ*. A good agreement in the shape of the distributions is observed, although the maximal size of epidemics is systematically higher in the EGOref sampled contact networks than in the friendship network, especially at large *β*/*μ*.

**Figure 6:**
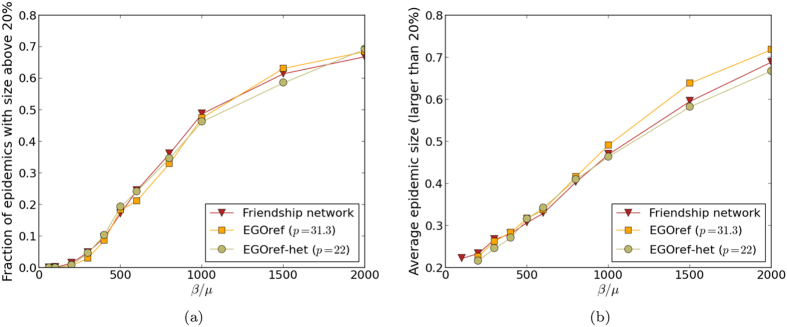
Outcome of SIR spreading simulations performed on friendship and both EGOref sampled networks. (**a**) Fraction of epidemics with size above 20% (at least 20% of recovered individuals at the end of the SIR process) as a function of the parameter of spreading *β*/*μ*. (**b**) Average size of epidemic with size above 20% as a function of the spreading parameter *β*/*μ*.

**Figure 7:**
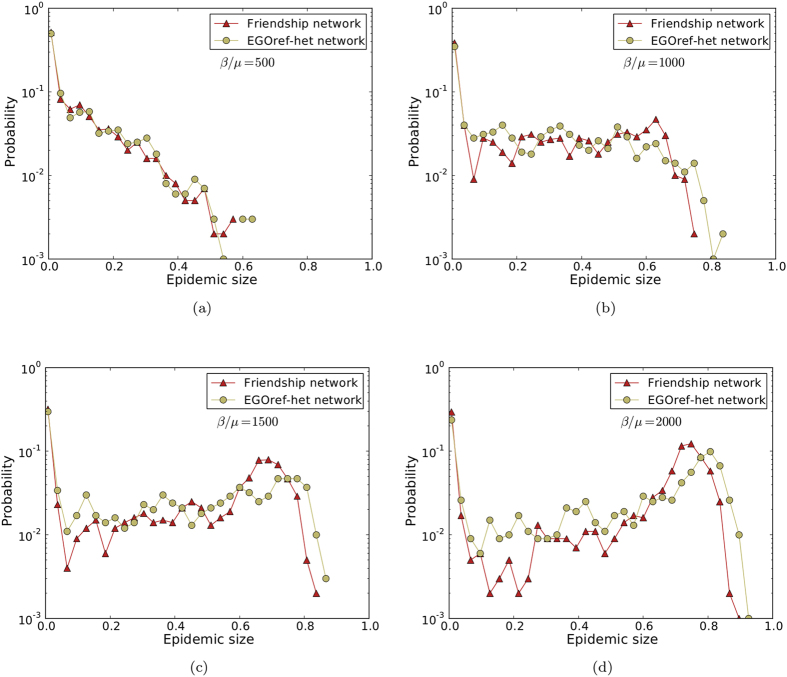
Comparison of the distributions of epidemic sizes of SIR spreading simulations performed on friendship and heterogeneous EGOref sampled networks for different values of *β*/*μ*.

We finally consider the EGOref-het sampling method. In that case, the number of sampled nodes is equal to the number of nodes of the friendship network in each class. We fix the number of edges to 413 by the same method as in the EGOref case; this is obtained for *p* ≈ 22. The resulting average shortest path length is 4.03 and the average clustering is 0.334, still smaller than in the friendship network and very close to the clustering in the EGOref sampled networks. We show in Figs 6 and 7 that the outcomes of the spreading simulations are similar to the EGOref case, with a slightly better agreement with the results of simulations using the friendship network, in particular for large epidemics.

### Sampling model exploration

In this section, we investigate how simulations of spreading processes performed on networks obtained from the contact network using the EGOref sampling method depend on the method’s parameters *p* and *N*. We consider 135 ≤ *N* ≤ 327 and 15 ≤ *p* ≤ 500 (as for *p *> 500 the number of links is almost equal to its maximum possible value). We first observe (not shown) that, at fixed *N*, the density of the sampled network is fully determined by the value of *p*. Changing *p* is thus equivalent to tuning the resulting network’s density. For *p* = 500 and *N* = 327, we almost recover the whole contact network.

**Figure 8:**
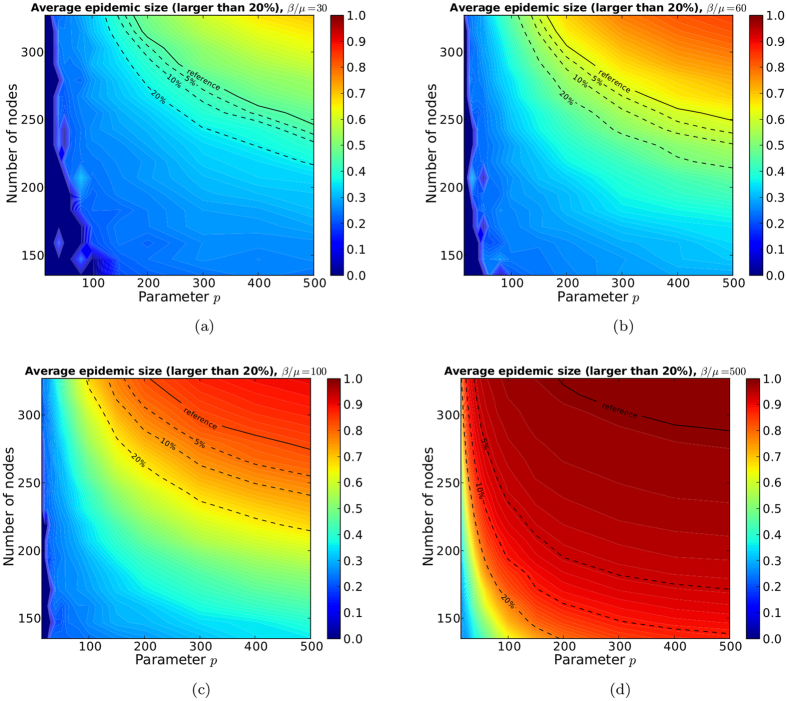
Color maps of the average epidemic size for epidemics with size above 20% for several values of *β*/*μ*. When no epidemics has size above 20%, the value is zero. The three dashed lines represent the value of average epidemic size at 5%, 10%, 20% of the reference value (solid line), which corresponds to the average epidemic size of the SIR spreading simulations performed on the contact network.

In Fig. 8, we show the average epidemic size obtained on the sampled networks as a function of *p* and *N* for different values of the spreading parameter *β*/*μ*: this size increases with both *p* and *N*. Increasing *p* (which determines the link reporting probability) at fixed *N* (the number of survey participants) or the contrary is not enough to obtain a correct estimation of the epidemic risk: both have to be increased in order to obtain the same value as when using the whole contact network, shown by the continuous line. The dashed lines show the values of *p* and *N* necessary to obtain an estimation of the epidemic size within 5%, 10% or 20% of this reference value.

We also note that the average epidemic size obtained for the largest values of *p* and *N* is actually larger than the reference, although the corresponding EGOref network is structurally almost the same as the contact network. This discrepancy stems from the fact that the edge weights are placed differently in both cases: weights are indeed assigned at random on the edges of the network obtained by the EGOref sampling procedure. We show in the Supplementary Information that a weight reshuffling on the contact network leads indeed to an increase in the size of the simulated epidemics.

We moreover show in the Supplementary Information the impact of the EGOref sampling on the outcome of SIR simulations for two other data sets corresponding to contacts in a conference and in offices, comparing it with the result of a simple population sampling. The underreporting of contacts as modeled by *p* is shown to have a very strong impact.

## Discussion

In this paper, we investigated if data coming from friendship surveys can be considered as similar to a sampling procedure on the contact network in a population, in the context of the estimation of the outcome of spreading processes. We focus here on infectious diseases for which contact networks are considered as a relevant proxy of transmission possibilities^4^. The rationale leading to this question comes from the quantitative comparison of friendship and contact networks collected within the same population, discussed in Mastrandrea *et al.*^11^ in the context of a high school: friendship and contact networks are indeed different, and many short contacts occur between individuals who are not friends; however, the longest contacts, which play an important role in potential propagation events, effectively correspond to friendship links, and the overall structure of the networks, in terms of interactions between different classes, are similar. Objectively measured contact networks are not always available, and friendship networks could be easier to obtain than contact networks in some situations. Moreover, surveys asking individuals about their friends might suffer less from memory biases than contact diaries. Understanding if and how friendship surveys could be used in models of spreading processes would thus be interesting^32^. Most importantly, framing the relation between friendship networks and actual contact networks as a sampling procedure might also help design and evaluate procedures to compensate for the resulting biases in the estimation of epidemic risks.

To make progresses in this direction, we have considered a publicly available data set combining, for the same set of individuals, a contact network measured by wearable sensors and a friendship network obtained by a survey. We have considered several ways of sampling the contact network and investigated their similarity with the friendship network with respect to simulations of epidemic spread. We have first considered a uniform random sampling of nodes, as the friendship network has much less nodes than the contact network. The RN method simply samples nodes at random and considers the resulting induced subgraph, while the EGO method is closer to a survey procedure, as it includes nodes chosen at random and all their contacts. In both cases, the obtained networks have much higher densities than the friendship network. As a consequence, the outcomes of epidemic spreading performed on these networks yield a much higher estimation of the epidemic risk than the friendship network. We have therefore considered sampling methods in which the density could be tuned, at fixed number of nodes. In the RE and RNref methods, we choose edges at random, which leads to a rather small clustering coefficient in the sampled networks and thus the resulting estimation of the epidemic risk is still higher than for the friendship network. These methods seem indeed too naive to recover the small scale structures and correlations of the friendship network.

Finally, we designed the EGOref sampling method, in a way to mimic a survey procedure in which nodes (egos) report on their friendships, under the assumptions that all reported friendships correspond to contacts, and that the probability that a contact corresponds to a friendship is larger for longer contacts. Note that this method is not aimed at reproducing exactly the friendship network (in which some links in fact do not correspond to contacts), but its goal is to produce a sampled network whose properties are close enough to the friendship network to lead to similar outcomes when used in simulations of spreading processes. The sampling method depends, at fixed number of egos, on a single parameter *p*, which determines the density of the resulting sampled network. In particular, for a uniform sampling of nodes, we can choose *p* to have the same density as the friendship network. At fixed density, this method allows us to recover a higher coefficient of clustering than the other sampling methods. Most importantly, simulations of spreading processes performed on the resulting sampled network yield an estimation of the epidemic risk in very good agreement with simulations using the friendship network, for a wide range of spreading parameters.

Some limitations of our approach are worth discussing. First, the EGOref method considers a uniform sampling of nodes. Choosing the *egos* with the same class distributions than in the friendship network leads to a slightly better agreement, as shown through the EGOref-het method. The clustering coefficient of the sampled networks is however still lower than in the empirical friendship network. More involved sampling procedures allowing to control the clustering might be sought, but would result in more complex and less intuitive sampling rules. The way we choose to assign the weights to edges in the sampled networks can also be discussed. On the one hand, the distribution of weights is known to be very robust in human contact networks, even in very different contexts such as schools or hospitals^6^,^28^, so that it seems natural to use the empirical distribution of weights, which can be taken from publicly available datasets, to assign weights to links obtained through surveys. On the other hand, a random assignment of weights destroys edge-weights correlations that can have an impact on the outcome of spreading simulations. We have considered such a random assignment as our goal is to compare the friendship network to a sampled contact network: when only friendship data is available, a natural way to perform simulations of epidemic spreading processes is indeed to assign weights at random to the friendship links. More accurate ways to assign weights in order to mimic the correlations linking the strengths and degrees of nodes in the contact network would be of great interest, but might depend on the context. We have also considered static networks, while real contact networks evolve over time. As mentioned above, this is based on a twofold rationale: as friendship data to not include temporal information, the comparison should be done with a sampling of a static weighted contact network; Moreover, for slow propagation timescales corresponding e.g. to flu-like illnesses, the precise dynamic of the contacts does not represent a crucial information^28^. In order to extend the comparison to faster processes, one should create surrogate timescales on the links similarly to ref. 23. Finally, our study is based on only one specific population. This is due to the current lack of datasets in which both contact and friendship networks are available. Further investigations in different contexts would be of great interest.

The sampling method we have proposed here depends on two parameters: the number of respondents *N* and the parameter *p* that allows to tune the amount of contacts reported. We have started here to study its impact on various data sets of contact networks in different contexts, and future work will focus on designing and evaluating methods to estimate the epidemic risk from incompletely and non-uniformly sampled data, such as the one described by Génois *et al.*^23^ for uniform population sampling, and to understand their efficiency and limits when the sampling parameters are varied.

## Methods

### Data

The data we consider was collected by the SocioPatterns collaboration in a French high school in Marseille during the week December 2nd–6th 2013, and subsequently analysed and made publicly available^11^. We use here two different data sets describing different types of relationships between students. The first corresponds to close face-to-face interactions between individuals equipped with wearable sensors. These contacts were measured for 327 students structured in 9 classes (called “classes préparatoires”) corresponding to different fields of study: 3 classes of Biology with respectively 37, 33 and 40 students, 3 classes of Mathematics-Physics with respectively 33, 29 and 38 students, 3 classes of Physics-Chemistry with respectively 44, 39 and 34 students. The participation rate reached 86.3% (there were overall 379 students in the 9 classes). The second type of data describes friendships relations between students. Students were asked to give the names of their friends within the high school. Over the 327 participants, only 135 students (41.3%) answered the survey. Students who were cited as friend of a respondent but did not answer the survey were removed from the data. The 135 participants to the survey are distributed in the 9 classes as following: 10, 20 and 28 participants in the 3 classes of Biology; 21, 3 and 7 participants in the 3 classes of Mathematics-Physics; 21, 10 and 15 participants in the 3 classes of Physics-Chemistry. A link is drawn in the friendship network between two students if at least one reported the other as friend.

### SIR simulations

To perform epidemic spreading simulations on the different networks, we need to take the link weights into account. The weight *W*_*ij*_ of the edge between nodes *i* and *j* of the contact network gives the total duration of the contacts that occurred between *i* and *j* during the total duration of the measure, *T*. We thus consider normalised weights *w*_*ij*_ = *W*_*ij*_/*T*: for a Susceptible node *i* in contact with an Infectious node *j*, the probability of becoming Infectious is 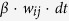 per time step *dt*. For an Infectious node, the probability of becoming Recovered during the time step *dt* is 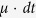. Each process starts with all nodes in the susceptible state except one infectious chosen at random (the seed). As we consider static networks, the parameters *β* and *μ* enter only through their ratio *β*/*μ* for the determination of the outbreak size. For each value of *β*/*μ*, results are averaged over 1000 simulations with randomly chosen seed. For the simulations on the friendship network, the weights are assigned at random to the links for each run. For the simulations on the sampled networks, a different sampling and weights assignment are performed for each run.

## Additional Information

**How to cite this article**: Fournet, J. and Barrat, A. Epidemic risk from friendship network data: an equivalence with a non-uniform sampling of contact networks. *Sci. Rep.* **6**, 24593; doi: 10.1038/srep24593 (2016).

## Supplementary Material

Supplementary Information

## Figures and Tables

**Table 1:** Features of the empirical networks and of the networks obtained with the simplest sampling methods: SubFr, RN, EGO preserving the number of nodes and RE preserving both the number of nodes and edges of the friendship network.

	Number of nodes	Number of edges	Density	Average degree	Average clustering	Avg shortest path*
Contact network	327	5818	0.11	35.6	0.503	2.15
Friendship network	135	413	0.05	6.1	0.532	4.06
SubFr	134	1235	0.14	18.4	0.546	2.22
RN	135	987	0.11	14.6	0.498	2.37
EGO	135	1679	0.19	24.9	0.573	2.04
RE	135	413	0.05	6.1	0.157	3.19

^*^The average shortest path length is computed on the largest connected component of the network.

**Table 2:** Features of the friendship network and of the sampled networks obtained by RE, RNref and EGOref method, all of them preserving the number of nodes and edges of the friendship network.

	Number of nodes	Number of edges	Density	Average degree	Average clustering	Avg shortest path*
Friendship network	135	413	0.05	6.1	0.532	4.06
RE	135	413	0.05	6.1	0.157	3.19
RNref	135	413	0.05	6.1	0.197	3.21
EGOref	135	413	0.05	6.1	0.355	3.90

^*^The average shortest path length is computed on the largest connected component of the network.
