# Supplementary material for: Epidemic risk from friendship network data: an equivalence with a non-uniform sampling of contact networks
Source: Sci Rep. 2016 Apr 15;6:24593. doi: 10.1038/srep24593 (PMC4832327; doi:10.1038/srep24593)
Supplement: Supplementary Information [file srep24593-s1.pdf]

Epidemic risk from friendship network data: an equivalence with a non-uniform sampling of contact networks. Supplementary Information

Julie Fournet[1], Alain Barrat[1,2,*]
[1] *Aix Marseille Université, Université de Toulon, CNRS, CPT, UMR 7332, 13288 Marseille, France*
[2] *Data Science Laboratory, ISI Foundation, Torino, Italy*
∗ *E-mail: alain.barrat@cpt.univ-mrs.fr*

1 Cases of the SubFr and the randomized friendship network

We show in Fig. S1 the outcome of SIR simulations performed on

- the SubFr network, obtained as the subgraph induced by the nodes who participated to the friendship survey on the contact network. This would correspond to a simple (non-uniform) population sampling of the contact network. Due to this population sampling, the nodes' degrees are underestimated and the outcome of spreading processes leads to smaller epidemic risk than when using the whole contact network. The difference is however rather contained with respect to the difference between contact and friendship networks.
- a randomized version of the friendship network using the algorithm of Maslov et al.[1]. In this case the number of nodes and links is exactly the same as in the friendship network, but structures and correlations are destroyed by the reshuffling. In particular, the clustering coefficient is much smaller, favoring the propagation. The obtained epidemic risk is higher than for the friendship network, but much smaller than for the contact network and the SubFr network.

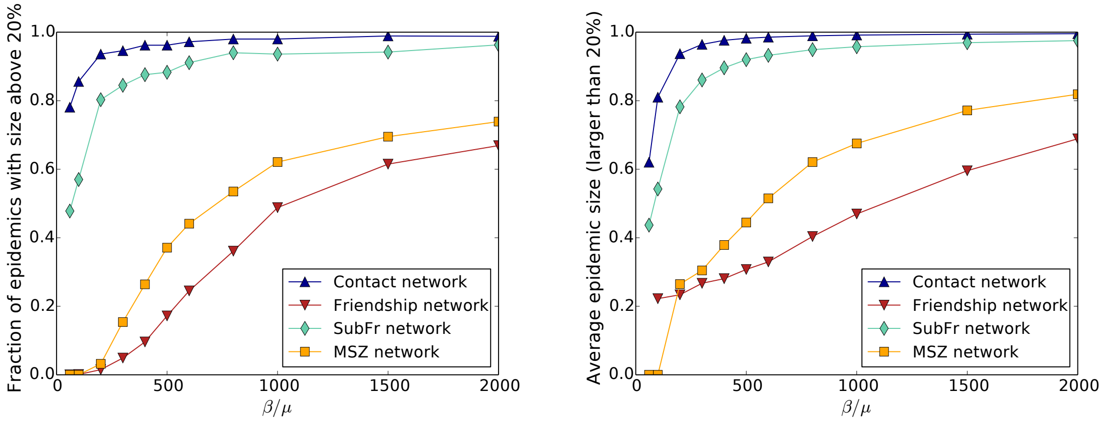

(a) (b)

Fig. S1: **Outcome of SIR spreading simulations performed on empirical, sampled and reshuffled networks.** We compare here the case of the SubFr sampling and of the randomized friendship network with the empirical contact and friendship networks. (a) Fraction of epidemics with size above 20% (at least 20% of recovered individuals at the end of the SIR process) as a function of β/μ. (b) Average size of epidemics with size above 20% as a function of β/μ.

2 Assigning weights to links

We investigate here the impact of several possible ways to assign weights to the networks used for the simulation of spreading processes, namely the empirical contact network measured by the wearable sensors, the network of reported friendships, and the networks obtained through various sampling procedures from the contact network. Indeed:

- the measured contact network is weighted, as the sensors give access to the duration of contacts. We can therefore consider the weighted network with its true weights ("Original weights") or, to assess the impact of correlations between weights and structure, reshuffle them at random among the network's links ("Reshuffled weights").
- any sampling procedure produces a subgraph of the contact network. As a consequence, the weights of the edges can be either taken directly from the contact network ("Original weights"), under the hypothesis that the sampling procedure keeps information about the weights. On the other hand, the opposite hypothesis, that sampling only informs about the existence of a link, and not on its importance, leads us to another weight assignment procedure, namely a random weight assignment from the distribution of weights of the contact network ("Random weights"). This is the procedure considered in the main text, as it is the most parsimonious and realistic in terms of availability of information.
- the friendship network is not weighted. In order to use it in the simulations of SIR process, we can assign weights to links in different ways:
  - we can choose the weights randomly from the distribution of weights in the contact network ("Random weights") or,
  - for each edge of the friendship network present in the contact network (86% of the links in the friendship network find a corresponding link in the contact network), we can use the corresponding weight and, for the remaining 14%, we can take the weights at random from the distribution of weights obtained from the first step (we call this assignment procedure "Original weights"), or,
  - we assign the weights as in the previous method and then reshuffle randomly the weights among the links of the friendship network ("Reshuffled weights").

2.1 Contact network and sampling procedures independent from weights

We note that methods of sampling in which edges are sampled independently from their weight (RE, RN, EGO) preserve the distribution of weights of the contact network. Moreover, reshuffling the weights in the contact network does not either change the distribution of weights.

Figures S2-S3 show however that simulations performed on the whole contact network with reshuffled weights leads to a larger epidemic risk evaluation than when the original weights are used. This result carries on to the case of RN, EGO and RE sampled networks: the use of randomly assigned weights systematically leads to larger epidemic sizes than the use of the real weights (Fig. S4). As the weight distribution is unchanged, this is the sign of the impact of some correlations between weights and structure.

Figure S5 shows that this is indeed the case: it displays the ratio s_k/k as a function of k for the contact network and the RN, EGO and RE sampled networks, where s_k is the average strength of nodes of degree k. When weights are shuffled or assigned at random, s_k/k is independent of k. For the original weights however, a distinct trend is observed, with smaller strengths at large k than for the reshuffled weights. As a consequence, the hubs have smaller spreading power than expected by random chance, and the epidemic spread is hindered, leading to smaller epidemic risk.

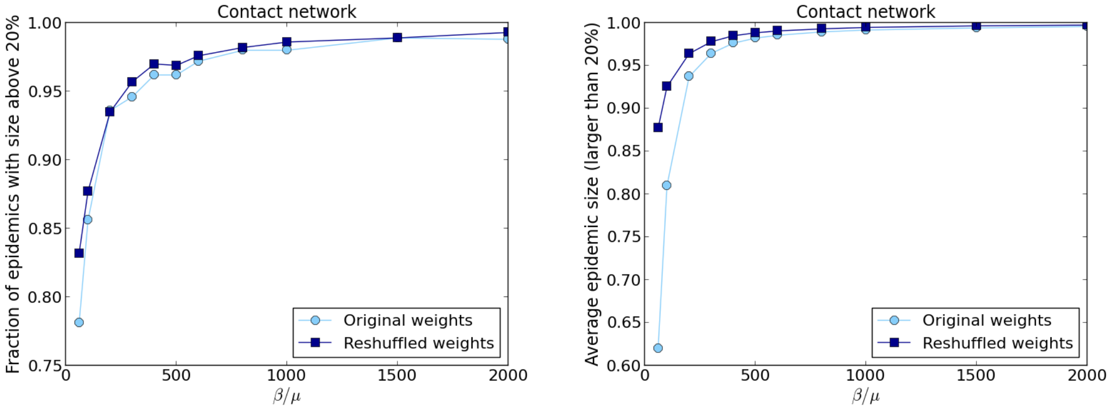

(a) (b)

Fig. S2: **Outcome of SIR spreading simulations performed on contact network with "Original weights" and "Reshuffled weights".** (a) Fraction of epidemics with size above 20% (at least 20% of recovered individuals at the end of the SIR process) as a function of β/μ. (b) Average size of epidemics with size above 20% as a function of β/μ.

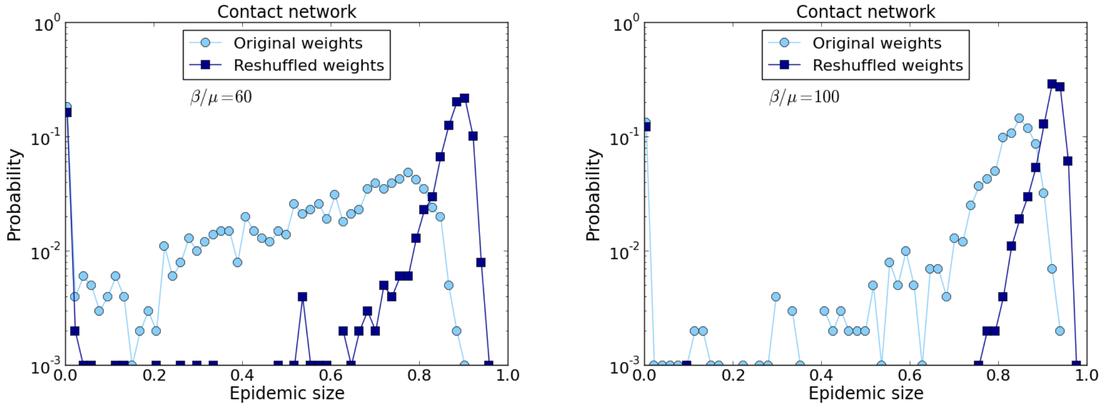

(a) (b)

Fig. S3: Comparison of the distributions of epidemic sizes of SIR spreading simulations performed on the whole contact network with "Original weights" and "Reshuffled weights" for two different values of β/μ.

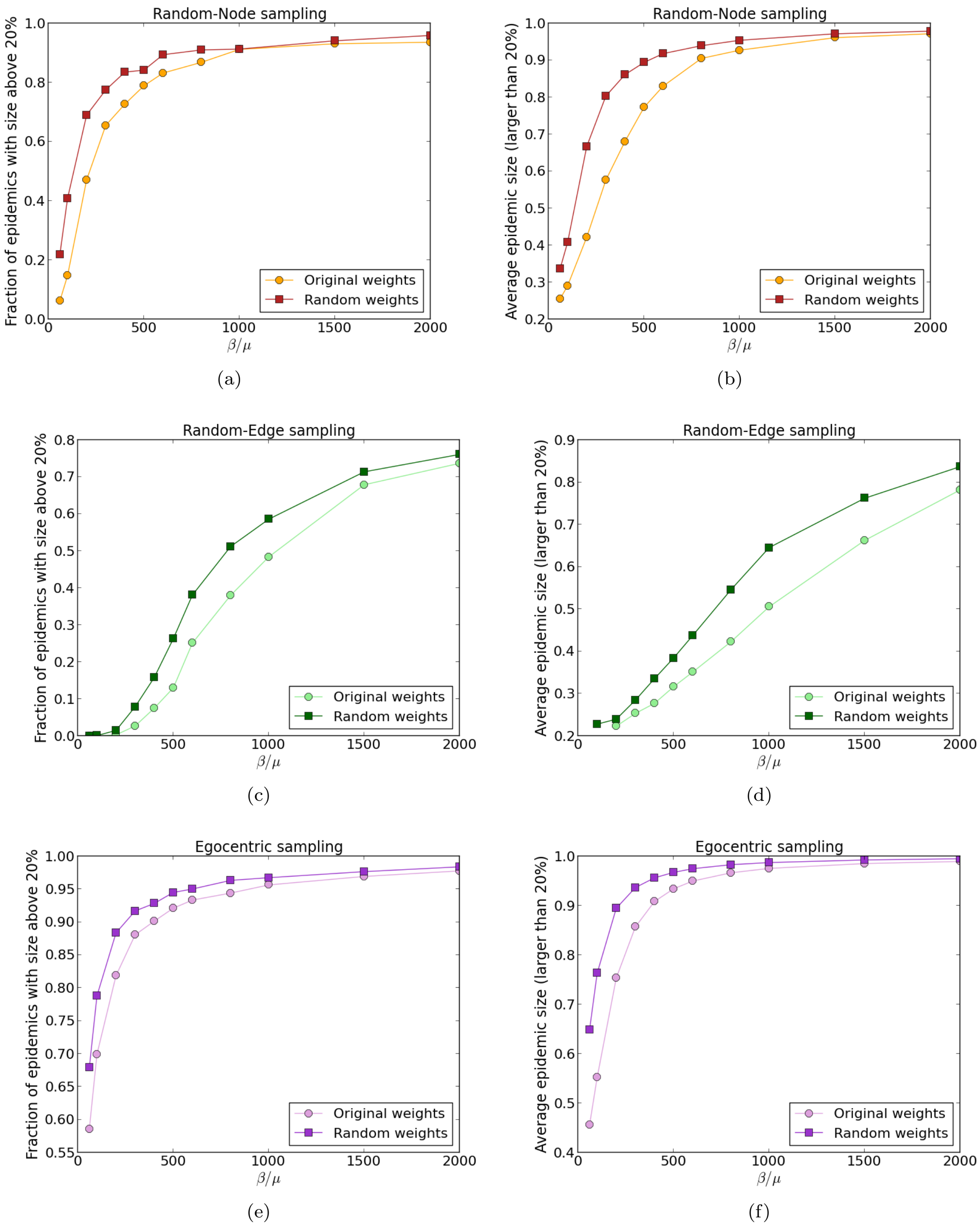

Fig. S4: **Outcome of SIR spreading simulations performed on contact networks sampled with the RN, RE and EGO methods, and with weights assigned either through the "Original weights" or "Random weights" procedures.** (a),(c),(e) Fraction of epidemics with size above 20% (at least 20% of recovered individuals at the end of the SIR process) as a function of β/μ. (b),(d),(f) Average size of epidemics with size larger than 20% as a function of β/μ.

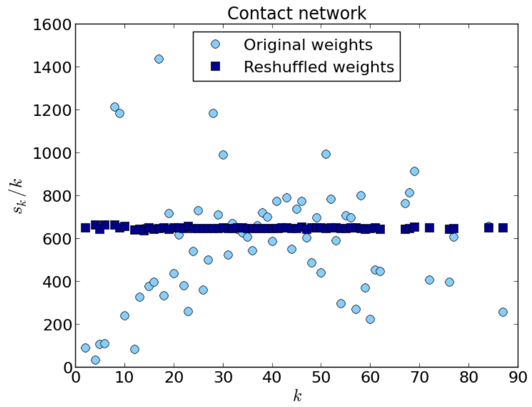

(a)

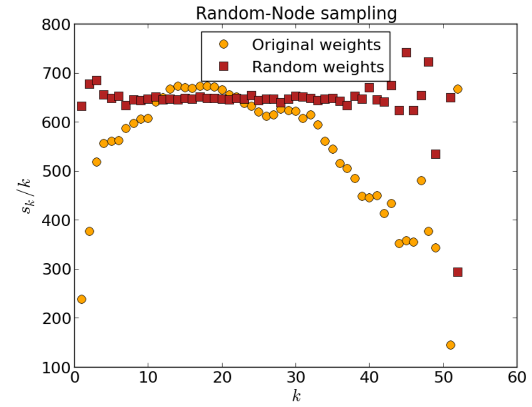

(b)

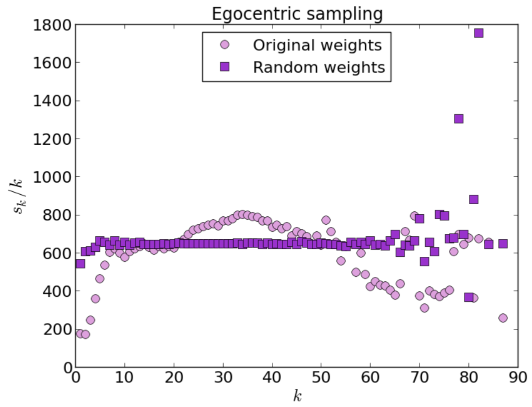

(c)

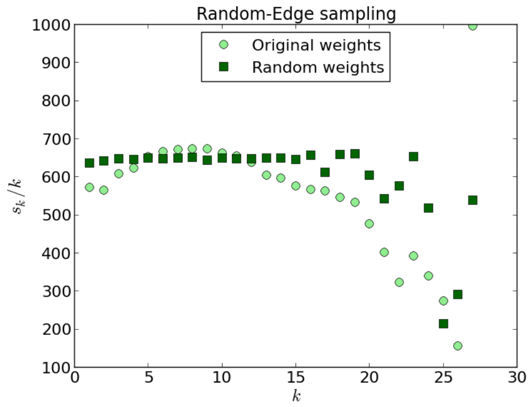

(d)

Fig. S5: Comparison of average strength s_k of nodes with degree k, divided by the degree k, as a function of k between the "Original weights" and the "Reshuffled/Random weights" cases for (a) the contact network, (b) the RN-sampled network, (c) the EGO-sampled network, (d) the RE-sampled network.

2.2 Friendship network

Figures S6 and S7 show the outcomes of SIR simulations on the friendship network with weights assigned in the three different ways described above. The size of epidemics is a little higher in the case of "Reshuffled weights" than in the case of "Original weights": this is due to the same mechanism as for the contact network, i.e., to correlations between weight and structure that are destroyed by the reshuffling.

Simulations on the network with "Random weights" lead on the other hand to a much smaller epidemic risk. As shown in Figure S8 and discussed in [2] indeed, the friendship links that are also present in the contact network tend to correspond to larger cumulative contact durations: the distribution of weights of the links present in both networks is not the same as the overall distribution of cumulative contact durations. Since the latter is used in the "Random weights" assignment procedure, the average weight is larger in the "Original weights" case, and this of course favours the spread.

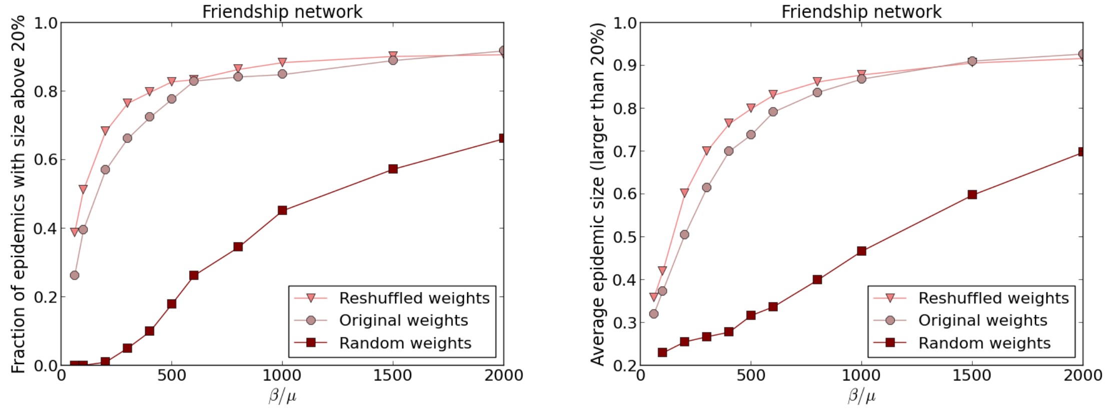

(a) (b)

Fig. S6: **Outcome of SIR spreading simulations performed on the friendship network with "Original weights", ' 'Reshuffled weights" and "Random weights".** (a) Fraction of epidemics with size above 20% (at least 20% of recovered individuals at the end of the SIR process) as a function of β/μ. (b) Average size of epidemic with size above 20% as a function of β/μ.

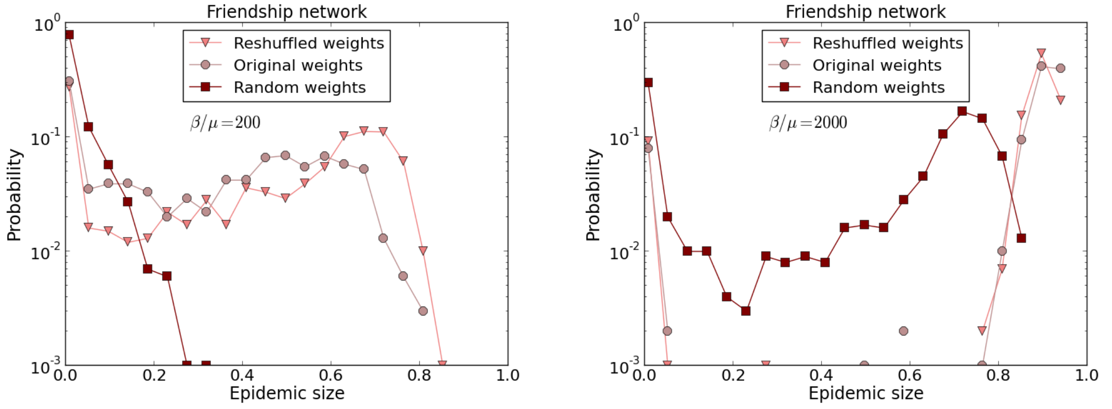

(a) (b)

Fig. S7: Comparison of the distributions of epidemic sizes of SIR spreading simulations performed on the friendship network with "Original weights", "Reshuffled weights" and "Random weights" for two different values of β/μ.

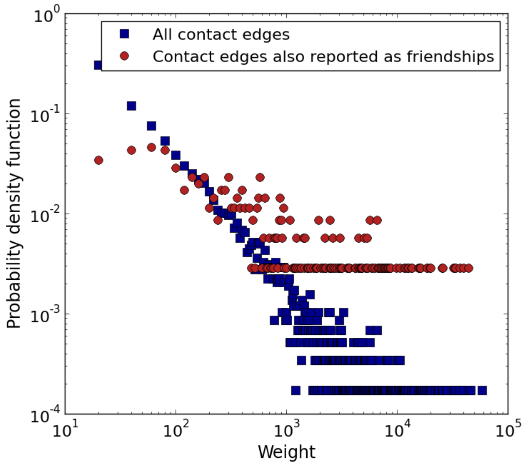

Fig. S8: Distribution of weights for different kinds of edges in the contact network : (i) all edges, (ii) edges corresponding to a reported friendship (i.e., present in both friendship and contact networks).

2.3 EGOref sampling procedure

The case of the EGOref sampling procedure is interesting as it combines the two effects discussed above, and the difference between the outcomes of simulations performed on networks using the "Original weights" and "Random weights" assignment procedures depends on the parameters of the sampling procedure p and N, as shown in Figure S9. For small p, the average epidemic size is higher in the case of "Original weights" whereas for large p, it is higher in the "Random weights" case. This can be explained by the two following competing effects:

- at small p, relatively few edges are selected in the contact network, and each ego selects preferentially links with large weights. The resulting distribution of original weights is thus biased towards large weights, and the weights are on average larger than when using weights taken at random from the overall distribution of weights of the contact network. This tends to favour the spread and thus leads to a larger epidemic risk for "Original weights" than for "Random weights".
- at large p, the probability to select an edge is large even for links with small weights. As a result, the distribution of weights of sampled links becomes close to the global distribution of weights in the contact network. The correlations between weights and structure present in the contact network can then play a role and act in the same way as for the RN, RE and EGO sampling methods: a random assignment of weights destroys the correlations and favours the spread.

We finally note that the value of N does not change the sign of the difference between the epidemic risk obtained by the two weight assignment procedures, but only its amplitude.

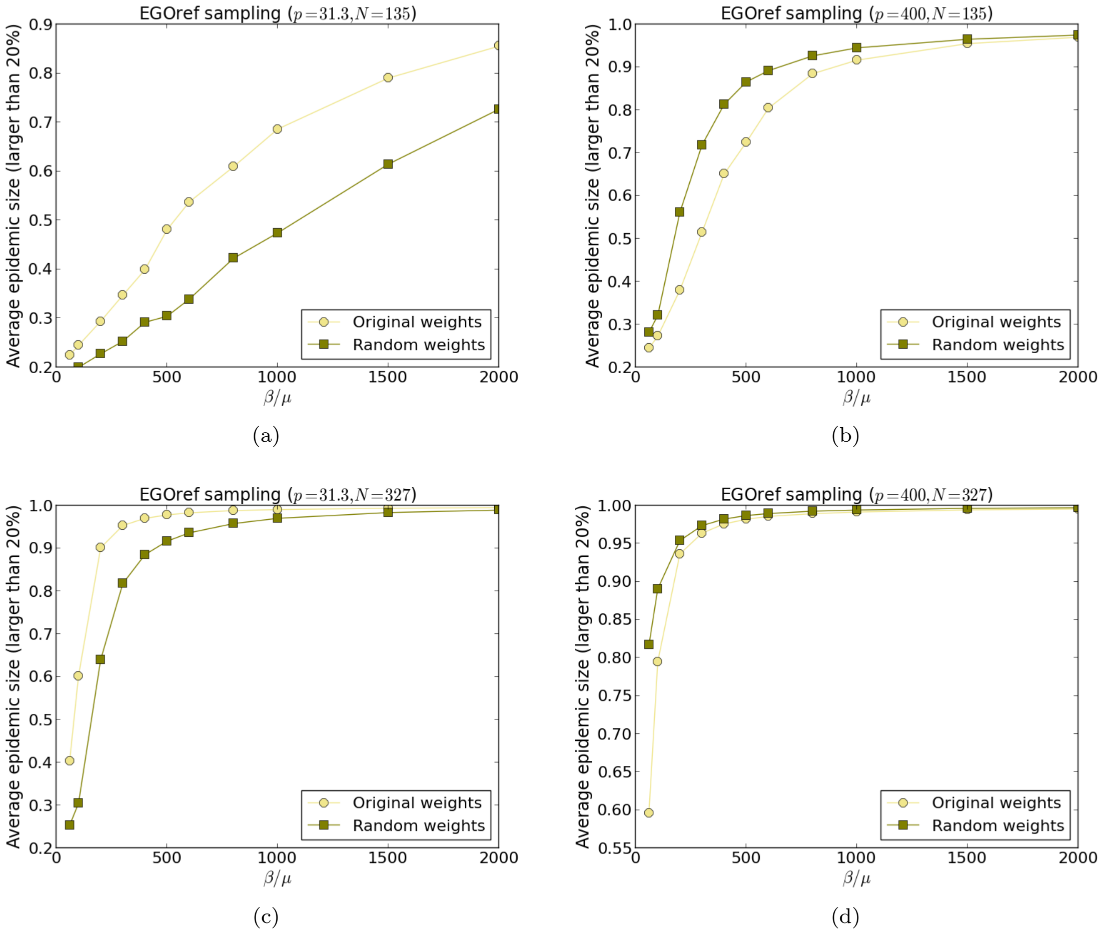

Fig. S9: **Average size of epidemic (with size above 20%) for SIR spreading simulations performed on EGOref-sampled network with "Original weights" and "Random weights" for four different couples of p and N :** (**a**) $p = 31.3, N = 135$, (**b**) $p = 400, N = 135$, (**c**) $p = 31.3, N = 327$, (**d**) $p = 400, N = 327$.

3 Impact of the EGOref sampling on other data sets

We consider here two data sets describing contacts in (i) a conference (SFHH) and (ii) offices (InVS). Both data sets were described for instance in [3]. The SFHH data describes contacts between 403 individuals during the two days of a conference, while the InVS data contains the contacts measured in offices during two weeks for 92 individuals. We first show in Fig.s S10-S13 the case of the same parameters as in the main text, namely the same value of p and the same fraction of nodes, i.e. 41%. We also show in these figures the outcome of simulations performed on a population sampling of the contact network with the same sampling fraction (RN sampling), in order to show separately the effects of population sampling (which keeps the density of the contact network fixed [3]) and of the selection of the links with probability proportional to their weights (EGOref mechanism).

We moreover show in Fig.s S14-S15 the equivalent of Fig. 8 of the main text for these two data sets, highlighting the combined effects of population sampling and of the absence of links with small weights in the sampled network.

	Number of nodes	Number of edges	Density
SFHH Contact network	403	9565	0.12
SFHH EGOref network	165	766	0.06
SFHH RN network	165	1598	0.12
InVS Contact network	92	755	0.18
InVS EGOref network	37	88	0.13
InVS RN network	37	119	0.18

Tab. S1: Number of nodes and edges in the empirical and sampled data sets. The sampled networks consider the same fraction of nodes as for the data set used in the main text.

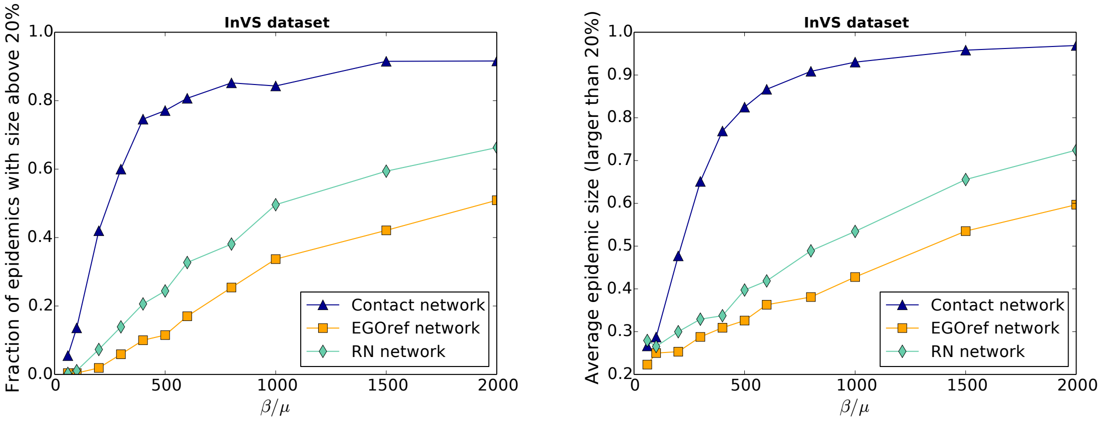

(a) (b)

Fig. S10: **Outcome of SIR spreading simulations performed on empirical and sampled contact networks (InVS data set).** We compare here the simulations on the original contact network with a sampled network using the EGOref sampling procedure with $p = 31.3$ and $N = 165$ nodes (corresponding to a sampling fraction equal to the case of the main text) and with the Random Nodes case (still with $N = 165$ nodes). (a) Fraction of epidemics with size above 20% (at least 20% of recovered individuals at the end of the SIR process) as a function of β/μ. (b) Average size of epidemics with size above 20% as a function of β/μ.

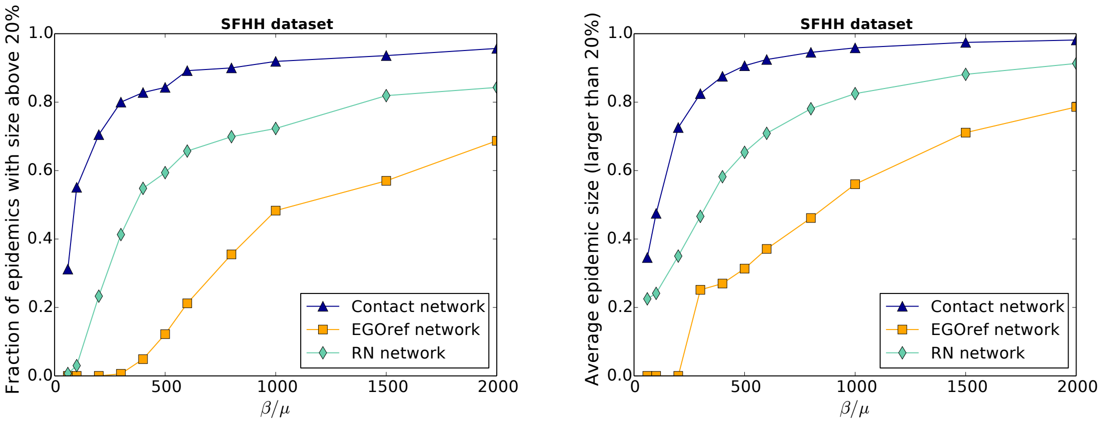

(a) (b)

Fig. S11: **Outcome of SIR spreading simulations performed on empirical and sampled contact networks (SFHH data set).** We compare here the simulations on the original contact network with a sampled network using the EGOref sampling procedure with $p = 31.3$ and $N = 37$ nodes (corresponding to a sampling fraction equal to the case of the main text) and with the Random Nodes case (still with $N = 37$ nodes). (a) Fraction of epidemics with size above 20% (at least 20% of recovered individuals at the end of the SIR process) as a function of β/μ. (b) Average size of epidemics with size above 20% as a function of β/μ.

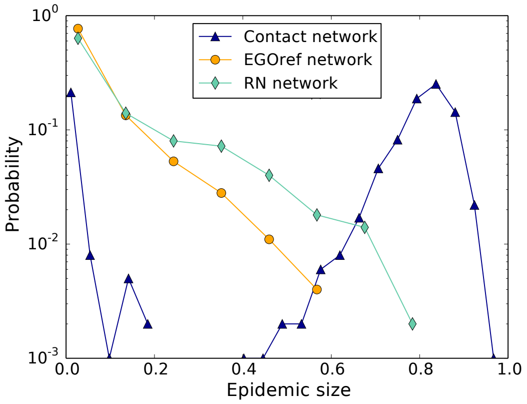

(a) $\beta/\mu = 500$

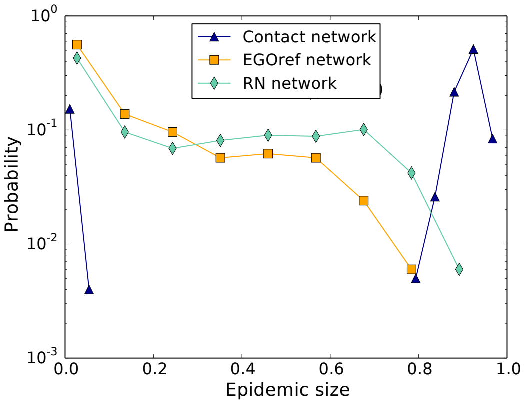

(b) $\beta/\mu = 1000$

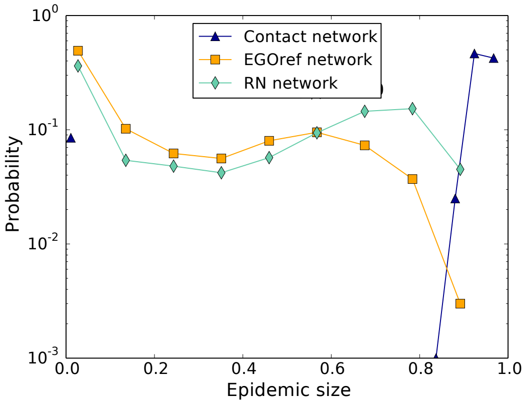

(c) $\beta/\mu = 1500$

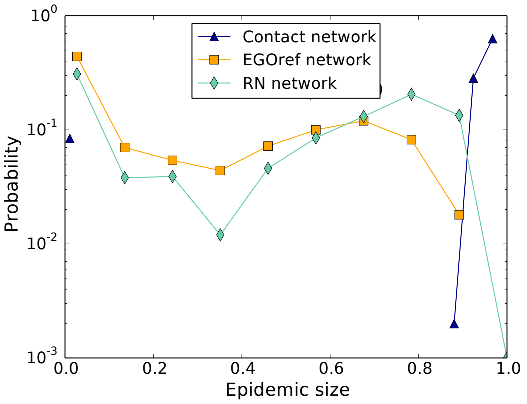

(d) $\beta/\mu = 2000$

Fig. S12: **Distributions of epidemic sizes of SIR spreading simulations (InVS data set).** We compare the distributions of epidemic sizes for simulations performed on the original contact network and on the sampled network using the EGOref sampling procedure with $p = 31.3$ and $N = 37$ nodes (corresponding to a sampling fraction equal to the case of the main text) and with the Random Nodes case (still with $N = 37$ nodes), for different values of β/μ.

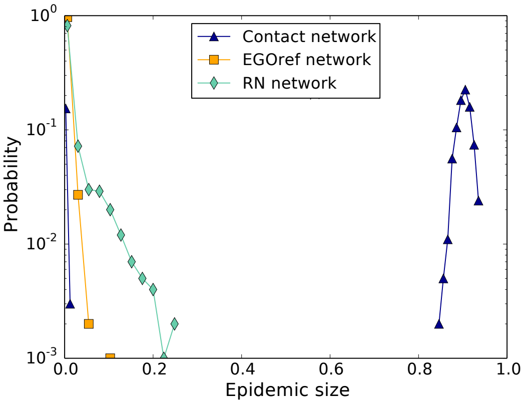

(a) $\beta/\mu = 500$

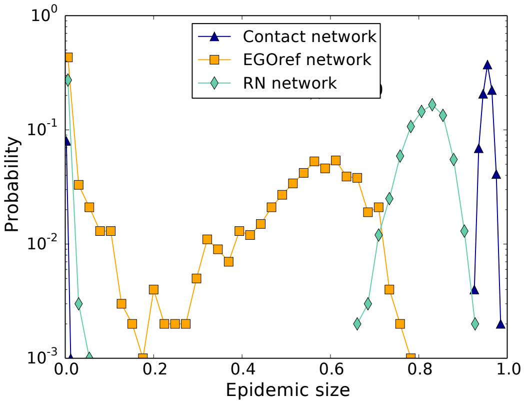

(b) $\beta/\mu = 1000$

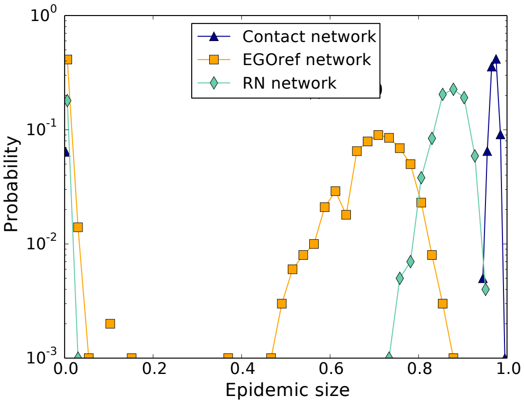

(c) $\beta/\mu = 1500$

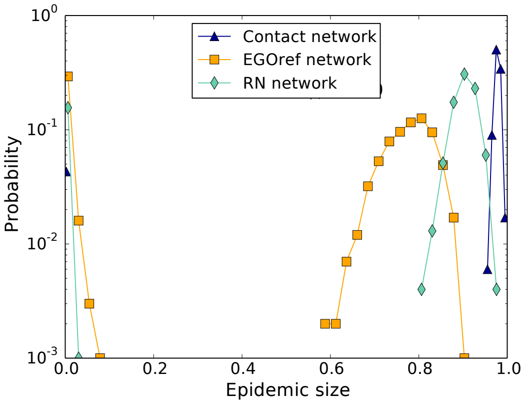

(d) $\beta/\mu = 2000$

Fig. S13: **Distributions of epidemic sizes of SIR spreading simulations (SFHH data set).** We compare the distributions of epidemic sizes for simulations performed on the original contact network and on the sampled network using the EGOref sampling procedure with $p = 31.3$ and $N = 165$ nodes (corresponding to a sampling fraction equal to the case of the main text) and with the Random Nodes case (still with $N = 165$ nodes), for different values of β/μ.

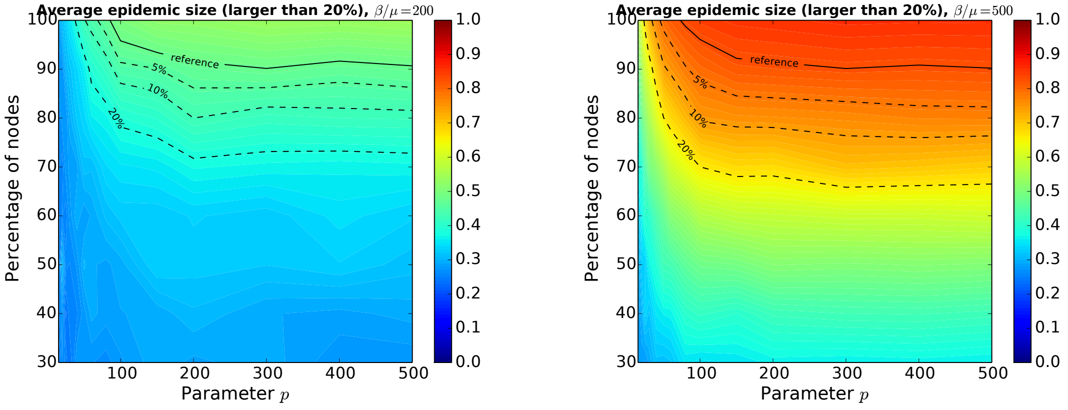

(a) (b)

Fig. S14: **Color maps of the average epidemic size for epidemics with size above 20% for several values of β/μ (InVS data set).** When no epidemics has size above 20%, the value is zero. The three dashed lines represent the value of average epidemic size at 5%, 10%, 20% of the reference value (solid line), which corresponds to the average epidemic size of the SIR spreading simulations performed on the contact network.

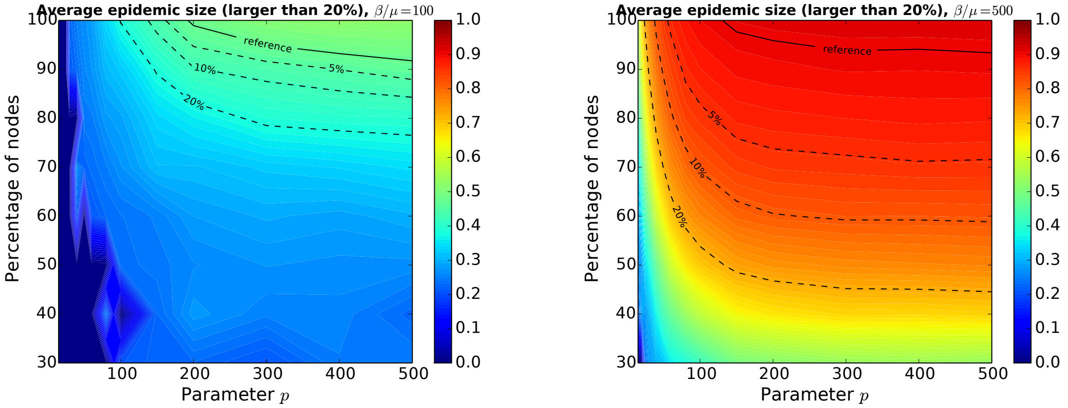

(a) (b)

Fig. S15: **Color maps of the average epidemic size for epidemics with size above 20% for several values of β/μ (SFHH data set).** When no epidemics has size above 20%, the value is zero. The three dashed lines represent the value of average epidemic size at 5%, 10%, 20% of the reference value (solid line), which corresponds to the average epidemic size of the SIR spreading simulations performed on the contact network.

References

[1] Maslov S, Sneppen K, Zaliznyak A (2004) Detection of topological patterns in complex networks: correlation profile of the Internet. Physica A 333, 529–540.

[2] Mastrandrea R, Fournet J, Barrat A (2015) Contact Patterns among High School Students. PLoS One 10: e136497.

[3] Génois M, Vestergaard C, Cattuto C, Barrat A (2015) Compensating for population sampling in simulations of epidemic spread on temporal contact networks. Nat. Comm. 6, 9860.
